# Knowledge, attitude, and practice among community pharmacists toward adverse drug reaction reporting and pharmacovigilance: A nationwide survey

**DOI:** 10.1016/j.rcsop.2025.100578

**Published:** 2025-02-18

**Authors:** Tahani Alwidyan, Mohannad Odeh, Ameerah Hasan Ibrahim, Eman Harahsheh, Aya Banat

**Affiliations:** a Department of Clinical Pharmacy and Pharmacy Practice, Faculty of Pharmaceutical Sciences, The Hashemite University, Zarqa, Jordan; b Department of Pharmacy, Faculty of Pharmacy, Al-Zaytoonah University of Jordan, Amman, Jordan

**Keywords:** Pharmacovigilance, Adverse drug reaction reporting, Community pharmacists, Knowledge, Attitude, Practice, Iatrogenic disease, Barriers

## Abstract

**Background:**

To enhance the impact of pharmacovigilance on drug safety, it must be integrated into the healthcare system. This study aimed to examine community pharmacists' knowledge, attitudes, and practices regarding adverse drug reaction reporting and pharmacovigilance.

**Methods:**

A self-administered, cross-sectional questionnaire study was conducted among eligible community pharmacists between July and September 2023. Participants completed online and paper-based questionnaires after providing prior consent. Descriptive and inferential analyses were performed using the Statistical Package for Social Sciences (SPSS, version 28). One-way ANOVA was used to assess the significance of the association between knowledge, attitude, practice scores, and demographic characteristics.

**Results:**

A total of 239 pharmacists completed the questionnaire (response rate of 67.5 %). Of them, 65.3 % were females. Poor knowledge regarding pharmacovigilance was evident in 66.1 % of participants, while only 11 % demonstrated good knowledge. Attitudes were primarily neutral (72.8 %), and practices were categorized as fair for 41.0 % and poor for 31.0 % of pharmacists. Significant factors influencing adverse drug reaction reporting included years of experience (*P* = 0.012) and awareness of the pharmacovigilance center (*P* = 0.000). The serious nature of adverse drug reactions was identified as the key facilitator for reporting (40.5 %), while well-documented adverse drug reactions in the literature (21.2 %) and lack of time (19.5 %) were the primary barriers among pharmacists.

**Conclusion:**

This study emphasizes the potential enhancement of adverse drug reaction reporting among community pharmacists by addressing poor knowledge, neutral attitudes, and barriers like time constraints. Targeted educational interventions and structured reporting frameworks are essential to enhance pharmacovigilance and ensure medication safety.

## Highlights

• A low level of adverse drug reaction reporting (ADR) activity was highlighted in this study
• This study identifies key barriers and enablers of ADR reporting, highlighting the need for research on effective interventions.
• Effective use of pharmacovigilance systems can be a key strategy to systematically enhance ADR reporting activity
• Policy changes are needed to integrate pharmacovigilance into routine clinical practice for better ADR monitoring and reporting.

## 1 Introduction

There is a global rise in the use of prescribed and non-prescribed medications.^1^ Alongside patient-related, drug-related, or socially-related factors, the risk of adverse drug reactions (ADRs) is increased.^2^ According to the World Health Organization, ADR has been defined as “*a response which is noxious and unintended, and which occurs at doses normally used in humans for the prophylaxis, diagnosis, or therapy of disease, or for the modification of physiological function*”.^3^ The negative impact of ADRs is well-known in the literature; several studies reported its consequences regarding cost,^4^^,^^5^ increased hospitalization,^6^ and mortality.^7^ Drug-induced diseases (iatrogenic diseases) are commonly encountered^8^ and referred to as ADR. In recent years, iatrogenic diseases have increased significantly and become a major global public health problem.^9^ Potential approaches to prevent and control ADR include proactive medication reviews, vigilance, and the close monitoring.

Pharmacovigilance is defined as the detection, assessment, understanding, and prevention of ADRs or other drug-related problems.^10^ Pharmacovigilance is crucial in optimizing drug safety and improving treatment outcomes. One review article published in 2019 reported the impact of pharmacovigilance programs in the withdrawal of dangerous medications (e.g., Cisapride) from the market.^11^ To ensure patient safety, several countries, including the United Kingdom, have implemented pharmacovigilance policies.^11^ However, the effectiveness of pharmacovigilance systems relies not only on the existence of policies but also on healthcare professionals' knowledge, attitudes, and practices (KAP) regarding ADR reporting. A lack of awareness, negative attitudes, and inadequate reporting practices among healthcare professionals have been identified as major challenges to pharmacovigilance implementation.^12^^,^^13^ Therefore, addressing these challenges is essential to improving ADR reporting and ensuring patient safety. The continuous increase in marketed drugs, the growing older population, polypharmacy, and emerging health problems could likely increase the occurrence of ADRs. Therefore, there may be an opportunity to increase ADR reporting activity in healthcare settings. Given that ADR reporting requires healthcare professionals to alter their behavior, interventions targeting behavior change are needed to promote ADR reporting. Behavioral analysis of healthcare professionals' knowledge, perceptions, enablers, and barriers to ADR reporting is the first step in identifying suitable components of future interventions.

Community pharmacists are the primary contact point for people to fulfil their daily healthcare needs; consequently, their role in pharmacovigilance systems is pivotal. In Jordan, community pharmacists have the authority to dispense medications, provide counseling, and ensure medication safety.^14^ However, their contribution to ADR reporting has been insufficiently explored. Although Jordan has a national pharmacovigilance system, studies indicate that ADR reporting activities in the country remain limited in scope and effectiveness.^15^, ^16^, ^17^, ^18^, ^19^ Among these studies, only two specifically assessed KAP^16^^,^^19^ and none utilized a nationwide scope, validated questionnaires, or demographic analysis to identify predictors of improved ADR reporting practices.

Similar studies have been conducted in Jordan across various healthcare settings emphasized the broader pharmacovigilance challenges. For example, Shroukh et al. (2018) examined pharmacovigilance knowledge and practices among physicians in Jordanian health centers, while Abu Hammour et al. (2024) analyzed pharmacovigilance data in a tertiary teaching hospital.^20^^,^^21^ These studies identified setting-specific barriers and emphasized the need to enhance training, awareness, and reporting systems to strengthen pharmacovigilance practices. However, comparable research focusing on community pharmacists remains scarce, particularly at the national level.

In the broader MENA region, previous studies have identified significant gaps in community pharmacists' KAP related to ADR reporting and pharmacovigilance.^13^^,^^22^^,^^23^ These include limited awareness of reporting systems, insufficient knowledge of pharmacovigilance processes, and low levels of practical engagement in ADR reporting. Additionally, perceived barriers such as time constraints and unclear reporting procedures that could hinder effective implementation have been identified.^13^^,^^22^^,^^23^ Further evaluation of pharmacovigilance systems, particularly in the context of ADR reporting, is essential to address these challenges.

This study aimed to evaluate the KAP of Jordanian community pharmacists regarding ADR reporting and pharmacovigilance. It also sought to identify barriers and enablers to guide development of future interventions to enhance pharmacovigilance activities within community pharmacies. By addressing these challenges, this study could improve ADR reporting in Jordan and the MENA region, where similar challenges persist.

## 2 Methods

### 2.1 Study design

A self-administered, cross-sectional questionnaire study was conducted in two phases among community pharmacists in Jordan. In the first phase, KAP questions were developed through a comprehensive literature review on ADRs and pharmacovigilance. A panel of eight pharmacy experts assessed the questions for relevance, followed by pilot testing with five community pharmacists and validation with 123 participants to ensure clarity, reliability, and content validity. Psychometric analysis demonstrated strong internal consistency (Cronbach's alpha = 0.772; composite reliability = 0.956). Further details about the questionnaire's development and validation are provided in Supplementary Material 1. In the second phase, the validated questionnaire was administered (online and paper-based) to eligible participants between July and September 2023.

### 2.2 Study participants and setting

Eligible participants included in this study were pharmacists with a minimum qualification of a BSc in Pharmacy or Doctor of Pharmacy, were working in a community pharmacy at the time of the study and were able to provide consent. This study was conducted in community pharmacies in Jordan. The main types of community pharmacies in Jordan are independent (pharmacist-owned) and pharmacy chains. Community pharmacies provide a wide range of prescription and nonprescription medications, skin care products, cosmetics, and medical equipment. The majority of patients seek medical advice from community pharmacists before visiting a physician due to ease of access.^24^

### 2.3 Sampling, recruitment and data collection

The sample size was estimated based on the formula for a finite population (N; Eq. (1)),^16^ considering the population size from the X Pharmacists Association website, a 95 % confidence interval (z = 1.96), and a 5 % margin of error (e = 0.05):

(1) $$\mathrm{Sa}m\mathrm{ple}\;\mathrm{size}\;\left(n\right)=\left[\;\frac{\left(\begin{array}{c} \frac{z^{2}p\left(1-p\right)}{e^{2}}\\ \; \end{array}\right)\;N}{\left(\frac{z^{2}p\left(1-p\right)}{e^{2}}\right)+\left(N-1\right)}\;\right]$$

Where:

*n* = Required sample size.

*N* = Population size.

*z* = *Z*-score (based on confidence level).

*p* = Estimated proportion of population.

*e* = Margin of error.

According to the Jordan Pharmacists Association website, there were 4437 community pharmacists in Jordan in mid-January 2023.^25^ Since it is a legal requirement in Jordan for each community pharmacy to employ at least one responsible pharmacist, the population size of community pharmacists was estimated to be 4437. Based on this population size, the required sample size was calculated to be 354. If a community pharmacy had more than one pharmacist, only one pharmacist was selected to participate, based on their availability and preference at the time of data collection.

A probabilistic stratified random sampling method^26^ was used to select community pharmacists in Jordan for this study. Stratification ensured the inclusion of community pharmacies from both urban and rural areas across different geographical regions in Jordan. This approach aimed to enhance the representativeness and diversity of the sample. A temporary list of community pharmacies, compiled from the Jordan Pharmacists Association website,^25^ included pharmacy codes and publicly available contact information (e.g., telephone, email, website). After randomization, the researcher (AB) contacted the selected pharmacies to verify pharmacist eligibility and obtain consent. Pharmacists were approached using two methods:

• Paper-based surveys: the researcher scheduled face-to-face visits at a convenient time and location. Before completing the questionnaire, participants signed a written consent form.
• Electronic surveys: a link to the online questionnaire, which included the consent form, was sent via email or messaging applications (e.g., WhatsApp, Skype, or Microsoft Teams).

Pharmacists who declined participation were thanked and excluded from further contact. Due to the anonymization of responses, no reminders were sent, which may have negatively impacted the response rate.

### 2.4 Study questionnaire

The study questionnaire consisted of five sections: (1) demographic and pharmacy-related information (7 questions); (2) awareness of ADR reporting and pharmacovigilance (8 questions); (3) knowledge about iatrogenic diseases (14 questions); (4) attitudes toward ADR reporting and pharmacovigilance, including perceptions of responsibility, confidence in reporting, and satisfaction with training (5 items on a 5-point Likert scale); and (5) practices related to ADR reporting, including the frequency of medication list reviews, ADRs documentation, and follow-up on reported incidents (4 multiple-choice questions). To further explore determinants of ADR reporting, two open-ended questions were included to identify barriers and enablers (Supplementary Material 2).

### 2.5 Data analysis

All data were coded and entered into a customized database developed using IBM SPSS® Version 28.0 (IBM, New York, USA) for statistical analysis. Descriptive statistics were reported for participant characteristics (e.g., age) and responses to questionnaire items (i.e., responses to Likert items). The Kolmogorov-Smirnov test was used to assess the normality of distribution for the variables. For categorical data, the Pearson Chi-square test was used to evaluate associations between study variables and the reporting of suspected ADRs. The KAP scores were compared across demographic variables (e.g., age, gender, university degree, and years of experience) and awareness-related variables (e.g., awareness about the pharmacovigilance center and the role of community pharmacists in ADR monitoring and reporting). The overall level of KAP was categorized using Bloom's cut-off point reference as follows:

• Knowledge: good (80–100 %), moderate (60–79 %), and poor (< 60 %).
• Attitude: positive (80–100 %), neutral (60–79 %), and negative (< 60 %).
• Practice: good (80–100 %), fair (60–79 %), and poor (< 60 %).

This method is commonly used to assess survey-based scores, ensuring standardized and objective categorization.^27^ One-way ANOVA was used to test for significant differences in continuous KAP scores among groups, with *p*-values and F-statistics reported. Despite minor deviations from normality, one-way ANOVA was used due to its robustness to moderate violations of this assumption.^28^ For all analysis, a p-value of <0.05 was considered statistically significant.

For open-ended questions, responses were analyzed using qualitative thematic analysis to identify key themes. Summarized findings highlighted barriers and enablers to ADR reporting. Participants responses were reviewed for logical inconsistencies, and where such discrepancies occurred (e.g., one participant claiming both no ADR reporting and reporting ADRs of 5 times and less, one participant claiming both no ADR reporting and telling the representative of the drug company about the ADR), these were retained as reported to preserve the integrity of the original responses.

### 2.6 Ethical approvals

Ethical approval was obtained for this study from the Hashemite University Institutional Review Board (reference number: 14/5/2022/2023) on 10th April 2023. Electronic or written informed consent was obtained from all subjects involved in the study.

## 3 Results

### 3.1 Participant characteristics

A total of 239 community pharmacists returned completed questionnaires out of 354 recruited during the study period, yielding a response rate of 67.5 %. This response rate is notably higher than similar studies conducted in Jordan. For instance, Abu Assab et al.^16^ reported a 63 % response rate,^16^ Alnawaiseh and Al-Oroud (2022) achieved 58 %,^19^ and Suyagh et al. (2015) reported 56 %.^29^ This may underscore the effectiveness of the recruitment process and enhances the reliability of our findings.

As illustrated in Table 1, most participants were female, accounting for 65.3 % (*n* = 156) of the sample, and a similar proportion (65.7 %, *n* = 157) fell within the age group of 21 to 30 years. The vast majority of participants (92.1 %, *n* = 220) were employed in urban pharmacies. Additionally, just under two-thirds (59.8 %, *n* = 143) had five years or less of experience in the community pharmacy sector.

**Table 1:** Demographics and community pharmacy-related information and association between dependent variable (reporting of suspected ADRs) and other variables (*n* = 239).

		Reporting of suspected ADRs			
Variable	Frequency (%)	Yes	No	χ^2^	P-value
Age					
21–30 years	157 (65.7)	77 (49)	80 (51)	5.0	0.175
31–40 years	55 (23)	36 (65.5)	19 (34.5)		
41–50 years	20 (8.4)	9 (45)	11 (55)		
51 years or more	7 (2.9)	4 (57.1)	3 (42.9)		

Gender					
Female	156 (65.3)	81 (51.9)	75 (48.1)	0.1	0.420
Male	83 (34.7)	45 (54.2)	38 (45.8)		

University degree					
Pharmacy	213 (89.1)	111 (52.1)	102 (47.9)	0.29	0.372
Doctor of pharmacy	26 (10.9)	15 (57.7)	11 (42.3)		

Qualification					
Bachelor's degree	223 (93.3)	115 (51.6)	108 (48.4)	2.1	0.335
Master's degree	15 (6.3)	10 (66.7)	5 (33.3)		
PhD degree	1 (0.4)	1 (100)	0 (0)		

Experience in community pharmacy practice					
One year or less	44 (18.4)	20 (45.5)	24 (54.5)	10.9	**0.012**
2–5 years	99 (41.4)	47 (47.5)	52 (52.5)		
6–9 years	44 (18.4)	33 (75)	11 (25)		
10 years or more	52 (21.8)	26 (50)	26 (50)		

Place of pharmacy					
Urban area (i.e., city, town)	220 (92.1)	119 (54.1)	101 (45.9)	2.1	0.114
Rural area (i.e., village)	19 (7.9)	7 (36.8)	12 (63.2)		

The area around the pharmacy					
Near a hospital	42 (17.6)	22 (52.4)	20 (47.6)	0.56	0.906
Near medical center	62 (25.9)	35 (56.5)	27 (43.5)		
Near medical clinic(s)	67 (28)	35 (52.2)	32 (47.8)		
No medical facilities around it	68 (28.5)	34 (50)	34 (50)		

Abbreviation: ADRs, adverse drug reactions.

The Chi-square test (χ^2^) was used to assess associations between reporting of suspected ADRs (dependent variable) and categorical demographic variables. Significant results are highlighted with corresponding *P*-values.

There was a statistically significant association observed between the reporting of suspected ADRs and the participants' years of experience. The distribution of reporting versus non-reporting was generally similar across all years of experience categories; however, a notable difference was observed in the 6–9 years' experience range, where 75 % of participants reported ADRs compared to 25 % who did not (*n* = 44, χ^2^ = 10.9, *P* = 0.012) (Table 1).

### 3.2 Awareness toward pharmacovigilance and ADR reporting practices

Table 2 shows that 62.3 % (*n* = 149) of participants accurately defined pharmacovigilance, while 27.2 % (*n* = 65) either gave incorrect answer (9.2 %, *n* = 22) or were unsure about the definition (18 %, *n* = 43). A substantial portion (45.6 %, *n* = 109) was unaware of the presence of a pharmacovigilance center for ADR reporting in Jordan. The majority of participants (*n* = 239) believed that ADRs should be monitored and reported by community pharmacists (82.4 % and 87 %, respectively). Any healthcare professionals (physicians, pharmacists, and nurses) were considered by more than half of the participants (57.3 %, *n* = 137) as the primary responsible parties for reporting ADRs, followed by physicians only at 14.2 % (*n* = 34). Among those who reported ADRs, more than two-thirds (67.5 %, *n* = 85) had reported ADRs five times or less during their professional careers. About 52.7 % (*n* = 126) of participants claimed they had reported ADRs, with informing a representative being the most common reporting method (25.9 %, *n* = 62). Participants who were aware of the presence of a pharmacovigilance center in Jordan tended to report suspected ADRs more frequently than those who were not aware (χ^2^ = 23.1, *P* = 0.000).

**Table 2:** Awareness of community pharmacists about ADR and pharmacovigilance reporting and association between dependent variable (reporting of suspected ADRs) and other variables (*n* = 239).

		Reporting of suspected ADRs			
Variable	Frequency (n, %)	Yes (n, %)	No (n, %)	χ^2^	P-value
Definition of pharmacovigilance					
The reporting of ADRs.	22 (9.2)	11 (50)	11 (50)	5.4	0.249
The detection, assessment, understanding, and prevention of ADRs.	149 (62.3)^a^	78 (52.3)	71 (47.7)		
I’m not sure but I could choose: the reporting of ADRs	15 (6.3)	12 (80)	3 (20)		
I'm not sure but I could choose: the detection, assessment, understanding, and prevention of ADRs	28 (11.7)	14 (50)	14 (50)		
I don't know.	25 (10.5)	11 (44)	14 (56)		

Awareness about the pharmacovigilance center					
Yes	130 (54.4)	87 (66.9)	43 (33.1)	23.1	**0.000**
No	109 (45.6)	39 (35.8)	70 (64.2)		

Awareness about the role of community pharmacists in monitoring ADR					
Yes	197 (82.4)	101 (51.3)	96 (59.5)	0.95	0.211
No	42 (17.6)	25 (48.7)	17 (40.5)		

Awareness about the role of community pharmacists in reporting ADR					
Yes	208 (87.0)	109 (52.4)	99 (47.6)	0.06	0.477
No	31 (13.0)	17 (54.8)	14 (45.2)		

qThe responsible person for reporting ADR is/are:					
Any healthcare professionals (physician, pharmacist, and nurse)	133 (55.6)	76 (57.1)	57 (42.9)	17.7	0.279
Physician	34 (14.2)	20 (58.8)	14 (41.2)		
Community pharmacist	21 (8.8)	10 (47.6)	11 (52.4)		
Clinical pharmacist	12 (5.0)	4 (33.3)	8 (66.7)		
Hospital pharmacist	5 (2.1)	3 (60)	2 (40)		
Physician, hospital pharmacist, clinical pharmacist and community pharmacist	4 (1.7)	3 (75)	1 (25)		
Any healthcare professional and patient	4 (1.7)	0 (0)	4 (100)		
Physician and hospital pharmacist	3 (1.3)	0 (0)	3 (100)		
Physician, hospital pharmacist, and community pharmacist	2 (0.8)	1 (50)	1 (50)		
Patients	2 (0.8)	1 (50)	1 (50)		
Nurse	1 (0.4)	0 (0)	1 (100)		
Physician and clinical pharmacist	1 (0.4)	0 (0)	1 (100)		
Clinical pharmacist, hospital pharmacist, and community pharmacist	1 (0.4)	1 (100)	0 (0)		
Community and clinical pharmacist	1 (0.4)	0 (0)	1 (100)		
Hospital and clinical pharmacist	1 (0.4)	0 (0)	1 (100)		
I do not know	14 (5.9)	7 (50)	7 (50)		

Suspected ADRs are reported in your community setting					
Yes	126 (52.7)	–	–	–	–
No	113 (47.3)	–	–	–	–

Number of events the participant reported ADRs					
Not applicable, I do not report ADRs	112 (46.9)	0 (0)	112 (100)	235.1	**0.000**
5 times and less.	86 (36)	85 (98.8)	1 (1.2)		
6–10 times.	23 (9.6)	23 (100)	0 (0)		
11–15 times.	7 (2.9)	7 (100)	0 (0)		
16–20 times.	3 (1.3)	3 (100)	0 (0)		
21 times and more.	8 (3.3)	8 (100)	0 (0)		

Method of ADR reporting					
Not applicable, I do not report ADRs.	11 (46.9)	0 (0)	112 (100)	235.1	**0.000**
I tell the representative of the drug company.	62 (25.9)	61 (98.4)	1 (1.6)		
I phone the drug company.	47 (19.7)	47 (100)	0 (0)		
I fill the adverse drug reaction reporting form.	9 (3.8)	9 (100)	0 (0)		
Tell the prescribing doctor	2 (0.8)	2 (100)	0 (0)		
Others, not specified	7 (2.9)	7 (100)	0 (0)		

Abbreviations: ADRs, adverse drug reactions.

The Chi-square test (χ^2^) was used to assess associations between awareness-related variables and ADR reporting. Significant results are highlighted with corresponding P-values.

a The number of participants who answered correctly about the definition of pharmacovigilance.

### 3.3 Enablers and barriers to ADR reporting

**Fig. 1:**
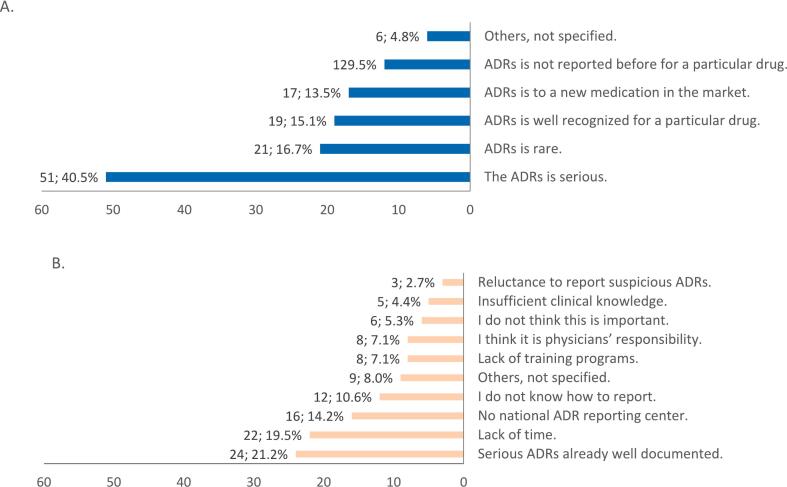
Enablers and barriers to adverse drug reactions (ADRs) reporting by the community pharmacist. A. Enablers of ADR reporting by the community pharmacist (*n* = 126). B. Barriers to ADR reporting by the community pharmacist (*n* = 113).

Fig. 1 provides an overview of the enablers and barriers to ADR reporting among community pharmacists. Among participants who reported ADRs (n = 126), five main factors facilitated their reporting practice. These factors were: the ADR is serious (40.5 %, *n* = 51), the ADR is rare (16.7 %, *n* = 21), the ADR is well recognized for a particular drug (15.1 %, *n* = 19), the ADR is associated with a newly marketed medication (13.5 %, n = 17), and the ADR was not previously reported for a particular medication (9.5 %, n = 12). Only 4.8 % of participants (n = 6) did not identify a reason that enabled their ADR reporting (Fig. 1A). Although 113 community pharmacists encountered one or more ADRs in their practice settings, none had reported a single ADR. Among them, 92 % (*n* = 104) provided reasons for not reporting. These reasons included: the serious ADR is well documented (21.2 %, *n* = 24), time constraints (19.5 %, *n* = 22), the absence of a national ADR reporting system (14.2 %, *n* = 16), lack of reporting skills (10.6 %, *n* = 12), lack of training programs (7.1 %, *n* = 8), the perception that ADR reporting is the responsibility of physicians (7.1 %, *n* = 8), the belief that ADR reporting is not an important issue (5.3 %, *n* = 6), insufficient clinical knowledge (4.4 %, *n* = 5), and reluctance to report suspicious ADRs (2.7 %, *n* = 3) (Fig. 1B).

### 3.4 Knowledge, attitudes, and practices related to ADRs and iatrogenic diseases

In Table 3A, the statement “*gastritis can be induced by taking nonsteroidal anti-inflammatory drugs*” had the highest proportion of “yes” responses (85.4 %, *n* = 204). Hypotension induced by ceftriaxone and psychosis induced by methylprednisolone both had the highest proportion of “no” responses (34.7 %, *n* = 83). The highest proportion of “I do not know” responses (43.5 %, *n* = 104) was observed for the statement concerning whether hyponatremia caused by carbamazepine can induce ischemic heart disease.

**Table 3:** knowledge, attitudes, and practices (KAP) responses of community pharmacists about iatrogenic disease, adverse drug reaction reporting and Pharmacovigilance (*n* = 239). A. knowledge, B. Attitude, C. Practice.

A. 14-item knowledge questions			
	Response, N (%)		
	Yes	No	I do not know
1-Gastritis can be induced by taking nonsteroidal anti-inflammatory drugs.	204 (85.4)	17 (7.1)	18 (7.5)
2- Paralytic ileus can be induced by taking loperamide.	114 (47.7)	57 (23.8)	68 (28.5)
3- Hypotension can be induced by taking ceftriaxone injection.	84 (35.1)	83 (34.7)	70 (29.3)
4- Hyponatremia leading to ischemic heart disease can be induced by taking carbamazepine.	96 (40.2)	38 (15.9)	104 (43.5)
5- Psychosis can be induced by taking methylprednisolone.	67 (28.0)	83 (34.7)	88 (36.8)
6- Cognitive dysfunction can be induced by taking prednisolone.	92 (38.5)	60 (25.1)	87 (36.4)
7- Parkinsonism can be induced by taking cinnarizine.	97 (40.6)	55 (23.0)	87 (36.4)
8- Obesity can be induced by taking risperidone.	155 (64.9)	32 (13.4)	52 (21.8)
9- Dyslipidemia can be induced by taking steroids (like estrogens and androgens).	131 (54.8)	59 (24.7)	49 (20.5)
10- Menstrual dysfunction can be induced by taking valproic acid.	125 (52.3)	51 (21.3)	63 (26.4)
11- Rhinitis can be induced by taking beta-blockers.	107 (44.8)	81 (33.9)	51 (21.3)
12- Pruritis can be induced by taking angiotensin-converting enzyme inhibitors.	81 (33.9)	70 (29.3)	88 (36.8)
13- Pruritis can be induced by taking statins.	81 (33.9)	76 (31.8)	82 (34.3)
14- Osteoporosis can be induced by taking methotrexate.	134 (56.1)	55 (23.0)	50 (20.9)

B. Attitude			
	Response, N (%)		
	Agree^a^	Disagree^b^	Neither agree nor disagree
1- I am satisfied that I have enough knowledge about iatrogenic diseases.	109 (45.6)	41 (17.2)	89 (37.2)
2- I am satisfied that I received sufficient education and training about iatrogenic diseases.	107 (44.8)	48 (20.1)	84 (35.1)
3- I am uncertain about recommending stopping the drug that I absolutely know its association with the reported problem by patient.	84 (35.1)	56 (23.4)	99 (41.4)
4- As a community pharmacist, I have a responsibility to report ADR to JFDA.	160 (66.9)	16 (6.7)	63 (26.4)
5- As a community pharmacist, I should only be required to consult the prescribing physician when a patient reports any problem associated to certain drug.	130 (54.4)	28 (11.7)	81 (33.9)

C. Practice			
	Response, N (%)		
	Always	Sometimes	Never
1- Do you review patient's medication list?	108 (45.2)	115 (48.1)	16 (6.7)
2- If patients tell you about symptoms occur with them, do you ask them about their medication list?	178 (74.5)	54 (22.6)	7 (2.9)
3- When a new medication introduced to the market, do you ask patients who are taking this medication if they experienced any side effect from it?	119 (49.8)	107 (44.8)	13 (5.4)
4- During your professional career, do you record the reported ADR from patients on patients' medical records?	65 (27.2)	102 (42.7)	72 (30.1)

Abbreviation: ADR, adverse drug reaction; JFDA, Jordan Food and Drug Administration.

a Sum of strongly agree and agree responses.

b Sum of strongly disagree and disagree responses.

As seen in Table 3B, the statement with the highest proportion of agreement responses (66.9 %, *n* = 160) was the one addressing the responsibility of reporting ADRs to community pharmacists. Conversely, the statement expressing uncertainty about stopping medication that causes the ADR by the community pharmacist had the highest percentage of disagreement, with 23.4 % (*n* = 56) of respondents indicating disagreement. Only 20.1 % (*n* = 48) of participants expressed satisfaction with the education and training received about iatrogenic diseases.

In Table 3C, a notable proportion of respondents (74.5 %, *n* = 178) selected always' in response to the question about asking for patients' medication lists when they report symptoms. The second highest proportion, 49.8 % (*n* = 119), selected “always” in response to the question about inquiring about side effects from patients taking newly introduced medications.

**Fig. 2:**
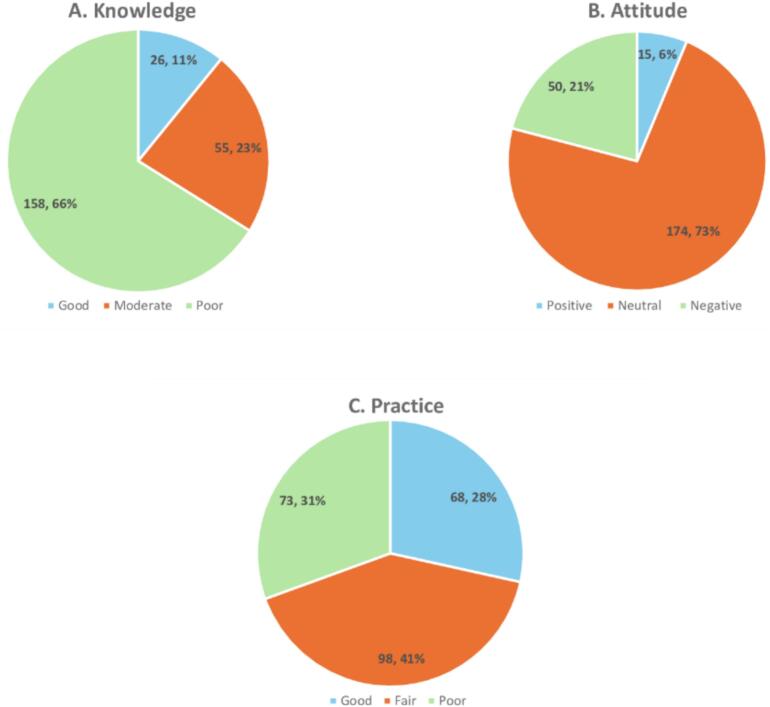
Distribution of KAP toward iatrogenic diseases ADR reporting and pharmacovigilance among community pharmacists (*n* = 239). **A.** community pharmacists were categorized based on their overall knowledge scores using the Bloom's cutoff points as “good knowledge” if a score ranges 80–100 % (11.2–14 points), “moderate knowledge” if a score ranges 60–79 % (8.4–11.1 point), and “poor knowledge” if a score ranges <60 % (<8.4 points). **B.** The overall level of attitude was categorized using Bloom's cut-off point, as a “positive attitude” if the score was 80–100 % (20–25 points), “neutral attitude” if the score was 60–79 % (15–19.9 points) and “negative attitude” if the score was less than 60 % (< 15 points). **C.** The total practice score was categorized using the Bloom's cutoff point, as “good practice” if the score was 80–100 % (6.4–8 points), ‘fair practice’ if the score was 60–79 % (4.8–6.3 points), “poor practice” if the score was <60 % (< 4.8 points).

The results of participants' knowledge, as illustrated in Fig. 2A, show the distribution across different categories. Specifically, 11 % (*n* = 26) of responders had a “Good” level of knowledge, 23.0 % (*n* = 55) had a “Moderate” level, and the majority, 66 % (*n* = 158), were categorized as having “Poor” knowledge.

The results presented in Fig. 2B indicate that a small percentage, 6 % (*n* = 6), expressed a “Positive” attitude. The majority of respondents, 73 % (*n* = 174), held a “Neutral” attitude, while 21 % (*n* = 50) reported a “Negative” attitude. These results highlight the need for efforts to improve positive attitudes among participants, as the majority have neutral or negative attitudes.

Fig. 2C displays the distribution of practice levels. It shows that 28 % (*n* = 68) of participants were categorized as having a “Good” level of practice, 41.0 % (*n* = 98) fell into the “Fair” category, and nearly one-third, 31 % (*n* = 73), were categorized as having a “Poor” level of practice. There is room for improvement in overall practice, as almost 70 % of participants fall into the “Fair” and “Poor” categories.

### 3.5 Factors influencing community pharmacists' KAP regarding ADR reporting

The overall mean knowledge score was 6.6 ± 3.9. Participants working in community pharmacies located near medical facilities had a significantly higher mean score (F (3) = 6.225, *P* = 0.000). Additionally, participants who were aware that community pharmacists have a role in monitoring and reporting ADRs had higher mean scores, with significant statistical differences (*P* = 0.001 and 0.008, respectively) (Table 4).

**Table 4:** Association of mean knowledge, attitude, and practice (KAP) score with study variables.

Variable	Knowledge score^a^				Attitude score^b^				Practice score^c^			
	mean ± SD	*P*-value	df	F- statistics	mean ± SD	P-value	df	F- statistics	mean ± SD	P-value	df	F- statistics
Age												
21–30 years	6.6 ± 3.8	0.924	3	0.159	15.8 ± 2.1	0.071	3	2.400	5.5 ± 1.7	**0.000**	3	6.800
31–40 years	6.7 ± 4.1				16.4 ± 2.1				6.1 ± 1.6			
41–50 years	6.6 ± 4.3				16.2 ± 2.0				4.3 ± 1.7			
51 years or more	5.6 ± 3.8				14.6 ± 1.9				4.3 ± 2.1			

Gender												
Female	6.6 ± 3.9	0.930	1	0.008	15.8 ± 2.2	0.174	1	1.860	5.5 ± 1.7	0.740	1	0.110
Male	6.5 ± 4.0				16.2 ± 1.9				5.6 ± 1.8			

University degree												
Pharmacy	6.4 ± 3.9	0.131	1	2.300	16.0 ± 2.1	0.240	1	1.400	5.5 ± 1.8	0.369	1	0.810
Doctor of pharmacy	7.7 ± 4.2				15.5 ± 2.2				5.8 ± 1.8			

Experience in community pharmacy practice												
One year or less	6.0 ± 3.9	0.710	3	0.461	15.7 ± 2.2	0.653	3	0.544	5.8 ± 1.8	0.138	3	1.860
2–5 years	6.7 ± 4.2				15.8 ± 2.0				5.6 ± 1.8			
6–9 years	6.9 ± 3.5				16.2 ± 2.1				5.7 ± 1.9			
10 years or more	6.5 ± 3.7				16.0 ± 2.1				5.0 ± 1.5			

The area around the pharmacy												
Near a hospital	7.3 ± 4.2	**0.000**	3	6.225	16.2 ± 2.0	0.092	3	2.171	5.7 ± 1.7	0.726	3	0.438
Near medical center	7.0 ± 3.8				16.0 ± 1.9				5.4 ± 1.9			
Near medical clinic(s)	7.3 ± 3.7				16.1 ± 2.3				5.6 ± 1.6			
No medical facilities around it	4.9 ± 3.6				15.4 ± 2.1				5.4 ± 1.8			

Awareness about the pharmacovigilance center												
Yes	6.7 ± 4.2	0.526	1	0.403	16.2 ± 1.8	**0.015**	1	5.970	5.8 ± 1.9	**0.001**	1	10.54
No	6.4 ± 3.5				15.6 ± 2.4				5.1 ± 1.5			

Awareness about the role of community pharmacists in ADR monitoring												
Yes	6.9 ± 3.9	**0.001**	1	10.363	16.2 ± 2.0	**0.000**	1	19.670	5.6 ± 1.8	0.131	1	2.291
No	4.8 ± 3.5				14.7 ± 2.0				5.1 ± 1.4			

Awareness about the role of community pharmacists in ADR reporting												
Yes	6.8 ± 3.9	**0.008**	1	7.105	16.0 ± 2.1	**0.039**	1	4.325	5.5 ± 1.8	0.823	1	0.050
No	4.8 ± 3.6				15.2 ± 2.0				5.6 ± 1.4			

Suspected ADRs are reported in your community setting												
Yes	6.4 ± 3.8	0.605	1	0.269	15.9 ± 2.0	0.924	1	0.009	5.7 ± 1.7	**0.045**	1	4.056
No	6.7 ± 4.2				15.9 ± 2.2				5.3 ± 1.8			

Abbreviations: ADR, adverse drug reaction.

One-way ANOVA was used to compare mean KAP scores across demographic and awareness-related variables. F-statistics and *P*-values are reported for significant findings.

a Mean knowledge score (±SD) = 6.6 (3.9).

b Mean attitude score (±SD) = 15.9 (2.1).

c Mean practice score (±SD) = 5.5 (1.8).

The overall mean attitude score was 15.9 ± 2.1. Participants who were aware of the pharmacovigilance system had a higher attitude mean score (16.2 ± 1.8) compared to those who were not aware (15.6 ± 2.4), with a significant statistical difference (F (1) = 5.970, *P* = 0.015). Furthermore, participants who were aware that community pharmacists have a role in monitoring and reporting ADRs had higher attitude scores, with significant statistical differences (*P* = 0.000 and 0.039, respectively) (Table 4).

The overall mean practice score was 5.5 ± 1.8. Participants aged between 31 and 40 years had a significantly higher mean score (6.1 ± 1.6) compared to other age groups (F (3) = 6.800, P = 0.000). Participants who reported suspected ADRs had a higher mean practice score (5.7 ± 1.7) than those who did not (5.3 ± 1.8), with a significant statistical difference (F (1) = 4.056, *P* = 0.045). Additionally, participants who were aware of pharmacovigilance across Jordan had a higher practice mean score (5.8 ± 1.9) compared to those who were not aware (5.1 ± 1.5), with a significant statistical difference (*P* = 0.001) (Table 4).

## 4 Discussion

### 4.1 Key findings: barriers and opportunities for ADR reporting

The findings of this study highlight the imperative need for effective and proactive strategies to enhance ADR reporting activities among community pharmacists. The optimal use of available pharmacovigilance systems could serve as a cornerstone strategy to systematically improve ADR reporting. Additionally, policy changes are required to integrate formal pharmacovigilance into routine clinical practice. This study aimed to assess pharmacists' KAP regarding ADR reporting and pharmacovigilance, and to identify the barriers and opportunities influencing its implementation in community settings. The study highlighted a low level of ADR reporting activity, which was significantly associated with pharmacists' awareness of the pharmacovigilance center, while other factors such as knowledge gaps and reporting barriers may also contribute.

In Jordan, community pharmacists are often the first point of contact for patients, particularly due to the ease of access to community pharmacies compared to hospitals or clinics.^30^ This places community pharmacists in a pivotal role for identifying and reporting ADRs. However, societal expectations and patient-pharmacist dynamics may influence pharmacists' confidence in reporting ADRs. For example, pharmacists may perceive that patients view them primarily as medication dispensers rather than as healthcare providers with a role in pharmacovigilance. Such societal attitudes could hinder proactive ADR monitoring and reporting behaviors.

While time constraints and workload have been identified as universal barriers across healthcare systems, pharmacy workloads in Jordan pose additional challenges.^31^ Community pharmacists often balance a range of responsibilities, including dispensing, patient counseling, and managing pharmacy operations.^14^ Unlike in hospital settings where ADR reporting systems are more formalized, community pharmacists often work independently, without institutional support or designated pharmacovigilance personnel. This variation highlights the need for workflow adjustments and strategies tailored to community pharmacy settings to mitigate the burden of ADR reporting.

The findings of this study are particularly relevant to Jordan but may also have broader applicability to other resource-limited or middle-income countries with similar healthcare systems. For example, countries where pharmacists operate in non-institutionalized settings or where pharmacovigilance frameworks are underdeveloped may face similar barriers to ADR reporting. By addressing factors such as pharmacist training, awareness, and system infrastructure, the strategies proposed in this study can inform initiatives in other contexts to optimize pharmacovigilance activities.

### 4.2 Interpretation and implications for clinical, policy changes and future research

This is one of the first studies to specifically examine the barriers and enablers of ADR reporting and pharmacovigilance among community pharmacists. Although not limited to pharmacists and conducted in diverse healthcare settings across different countries, several quantitative and qualitative studies have assessed healthcare professionals' perspectives on ADR reporting and pharmacovigilance, including the identification of barriers and enablers.^15^, ^16^, ^17^, ^18^, ^19^^,^^22^^,^^32^, ^33^, ^34^, ^35^, ^36^, ^37^, ^38^, ^39^, ^40^ The results of these studies were broadly comparable to the findings of the present study. One key similarity is the lack of time, which emerged as a significant challenge to ADR reporting. This has been consistently reported across studies in different settings, such as Pakistan,^32^ Vietnam^40^ and Saudia Arabia,^22^ where researchers reported that time constraints due to heavy workload hindered spontaneous reporting. This highlights the universal nature of this barrier across healthcare systems.

In addition, the present study identified a lack of knowledge about ADR reporting systems as key barriers. This observation is consistent with studies conducted in Jordan^18^ and Ghana,^38^ where healthcare professionals expressed uncertainty about ADR reporting procedures and insufficient familiarity with pharmacovigilance systems. These findings emphasize that improving awareness about pharmacovigilance systems is critical to overcoming this barrier. The uncertainty about how and where to report an ADR, identified as key barriers in the present study, were also highlighted in three previously published systematic reviews on ADR reporting and pharmacovigilance barriers and enablers.^41^, ^42^, ^43^ Two of these reviews examined healthcare professionals' ‘perspectives on these issues in a country-based manner.^42^^,^^43^ Consistent with the present study, these reviews identified key barriers such as healthcare professionals’ reluctance to report suspicious ADRs, lack of training programs, lack of confidence, and uncertainty about the responsible person to report ADRs.^41^^,^^43^

On the other hand, some differences were observed between the present study and findings reported in previous studies,^33^^,^^35^^,^^36^^,^^39^ which may attribute to cultural, systemic, and contextual factors. For example, while this study focused specifically on community pharmacists, research conducted in hospital or tertiary care settings highlighted the role of institutional policies and formal pharmacovigilance cultures in influencing reporting behaviors.^33^ Hospital-based healthcare professionals often benefit from stronger supervision and accountability, which may not be as prominent in community pharmacy settings where pharmacists operate more independently. While, the underreporting of ADRs due to fear of legal or professional consequences identified as a significant barrier in a previous study conducted in Pakistan,^36^ this barrier was not as prominently observed in the present study. This may suggest a potential differences in professional norms and reporting cultures among community pharmacists.

Further, resource limitations have also contributed to variations in findings across studies. For example, studies conducted in low- and middle-income countries, such as Vietnam^40^ and Jordan,^19^ highlighted the lack of digital tools, centralized reporting platforms, and other resource constraints as significant barriers. In contrast, the present study did not identify resource limitations to the same extent, which may reflect improvements in infrastructure within the study region over time.

Consistent with previous studies,^22^^,^^44^^,^^45^ the findings of this study showed that a substantial proportion of community pharmacists were well-versed in the definition of pharmacovigilance. In contrast, a previous cross-sectional study conducted among 135 healthcare professionals' in Saudi Arabia reported a low level of knowledge about pharmacovigilance terminology among participating pharmacists.^46^ Incorporating pharmacovigilance subjects into undergraduate curricula may enhance understanding, knowledge, and awareness of ADR reporting and pharmacovigilance.^47^

Although not in a community setting, a questionnaire-based survey study in Malaysia assessed pharmacists' perspectives on the barriers and enablers of ADR reporting and pharmacovigilance, revealing that factors such as well-documented ADRs and insufficient clinical knowledge could hinder ADR reporting. Conversely, serious, unusual ADRs, or those related to new or previously unreported drugs were considered factors that enhanced pharmacists' reporting.^34^ These findings align with the present study.

Most community pharmacists in this study (66 %) were found to have poor knowledge about iatrogenic diseases, whereas only 11 % had positive knowledge. These findings suggest that community pharmacists' knowledge about iatrogenic diseases is generally unfavorable. To our knowledge, no previous studies have examined knowledge about iatrogenic diseases among community pharmacists. However, the findings from this study highlight the need to incorporate iatrogenic disease reporting into pharmacy curricula and utilize educational channels to integrate iatrogenic disease reporting concepts into community pharmacists' careers, potentially altering their perception and attitude toward iatrogenic disease reporting.

More than two-thirds of the reporter participants in this study had reported only up to five ADRs during their work experience. Similar to our findings, previous studies have documented a low level of ADR reporting, with the percentage of reporting community pharmacists varying from 3 % to 34 % compared to our findings of 52.7 %.^13^^,^^23^^,^^46^ This variation may be attributed to the significant number of community pharmacists who were unaware of the presence of a pharmacovigilance center for ADR reporting. Continuing medical education and training programs in pharmacovigilance, spontaneous reporting, and incentives could help minimize underreporting. Countries like Malaysia^39^ and the United Arab Emirates^33^ provide examples of how robust reporting frameworks and support systems can facilitate ADR reporting among pharmacists.

Predictors related to pharmacists' KAP toward ADR reporting and pharmacovigilance were also explored in this study. The findings revealed that community pharmacists' awareness of the pharmacovigilance center significantly impacted their attitudes and practices toward ADR reporting. Community pharmacists who are aware of their role in ADR monitoring and reporting exhibited significantly better knowledge and attitudes toward ADR reporting and pharmacovigilance. Furthermore, good ADR reporting practices among community pharmacists were significantly associated with reporting suspected ADRs in their workplace. These findings suggest that participants with better awareness of the healthcare professional role in the pharmacovigilance process might have better opportunities to access updated information, improving their knowledge, attitude, and practice regarding ADR reporting and pharmacovigilance.

With limited pharmacovigilance guidelines and insufficient training programs, more structured guidance is needed to prevent medication harm. This study provides critical insights that can inform clinical practice, policy changes, and future research. Clinically, community pharmacists, as the first point of patient contact, play a pivotal role in ADR identification and reporting. However, their heavy workloads and lack of institutional support remain significant challenges. Addressing these barriers through workflow adjustments and tailored training programs focused on ADR reporting procedures and pharmacovigilance systems can strengthen ADR reporting practices. On a policy level, integrating formal pharmacovigilance frameworks in Jordan is essential. This includes developing clear ADR reporting guidelines, implementing mandatory training programs within undergraduate pharmacy education, offering continuing professional development workshops for practicing pharmacists, and initiating the role of a Medication Safety Officer for community pharmacies in Jordan. While this study did not evaluate specific educational interventions or strategies to improve pharmacovigilance practices among community pharmacists, the findings provide a solid foundation for their development. The identified gaps in pharmacists' KAP emphasize the need for structured training programs focusing on ADR reporting systems, practical pharmacovigilance skills, and iatrogenic disease recognition. Future research should include in-depth qualitative studies (e.g., interviews and focus groups) with pharmacists and other stakeholders to design tailored educational interventions. Additionally, evaluating the impact of these interventions on pharmacists' KAP and exploring the role of healthcare infrastructure and institutional support will provide actionable strategies, particularly in resource-limited settings such as Jordan.

### 4.3 Strengths and limitations

The validity and reliability measures of the questionnaire enhanced the quality and accuracy of the data collected. Despite ongoing ADR reporting activities, the current findings highlight areas for improvement, such as the need for policy changes to integrate formal pharmacovigilance practices into routine clinical activities in community settings. However, some limitations should be considered when interpreting the results. First, the cross-sectional design of this research makes it difficult to establish causal relationships. Second, the self-reported nature of the data collection introduces the possibility of recall bias, as pharmacists may have adjusted their responses to provide what they perceive as optimal answers. To minimize these biases, the study employed an anonymous questionnaire and assured participants of confidentiality, thereby enhancing the reliability and validity of the self-reported data. Third, the presence of logical inconsistencies in a small number of self-reported responses. For example, some participants reported never submitting an ADR while also indicating ADR reporting “*5 times or less*” or informing a drug representative. These inconsistencies likely resulted from participant misinterpretation of the questions or errors in self-reporting. Fourth, the final sample size (*n* = 239) was below the target of 354, representing a response rate of 68 %. This may have impacted the study's statistical power and introduced non-response bias, affecting precision and generalizability. Fifth, while the sample broadly reflected the expected demographic and professional distribution, minor deviations occurred due to factors such as pharmacist availability or willingness to participate. For example, variations in pharmacy location (urban vs. rural) and years of experience may have influenced responses. Sixth, this study did not collect data on the type of pharmacy (independent versus chain), which could influence pharmacists' workload and ADR reporting practices. Future studies should explore this variable to better understand its impact on pharmacists' characteristics and behavior regarding ADR reporting. Finally, since the questionnaire was administered exclusively to community pharmacists, it remains uncertain whether the findings are applicable to pharmacists in other healthcare settings, such as hospitals.

## 5 Conclusion

This nationwide study identifies significant gaps in community pharmacists' KAP toward ADR reporting and pharmacovigilance in Jordan. Pharmacists demonstrate fair reporting practices; however, poor knowledge and neutral attitudes present major challenges. Key barriers, such as lack of time, inadequate training, and unawareness of reporting systems, limit effective ADR reporting. To overcome these challenges, authorities must establish obvious ADR reporting guidelines, integrate mandatory pharmacovigilance education into undergraduate pharmacy curricula, and provide continuous professional development programs. Assigning a designated Medication Safety Officer for community pharmacies can enhance ADR reporting and integrate pharmacovigilance within routine workflows. Future efforts should prioritize policy changes, targeted educational interventions, and improved system infrastructure to strengthen ADR monitoring and promote medication safety in community pharmacy settings.

## Funding

This research did not receive any specific grant from funding agencies in the public, commercial, or not-for-profit sectors.

## Data access statement

All authors had complete access to the study data that supported the publication.

## CRediT authorship contribution statement

**Tahani Alwidyan:** Writing – review & editing, Writing – original draft, Validation, Methodology, Formal analysis, Data curation, Conceptualization. **Mohannad Odeh:** Writing – review & editing, Formal analysis. **Ameerah Hasan Ibrahim:** Writing – review & editing, Formal analysis. **Eman Harahsheh:** Writing – review & editing, Writing – original draft. **Aya Banat:** Writing – review & editing, Methodology.

## Declaration of competing interest

The author(s) declare that there are no conflicts of interest.

## Data Availability

The data underlying this article cannot be shared publicly to protect the privacy of individuals who participated in the study. The data will be shared on reasonable request to the corresponding author.

## Appendix A Supplementary data

Supplementary material 1 Image 1

Supplementary material 2 Image 2
