# Supplementary material for: Knowledge, attitude, and practice among community pharmacists toward adverse drug reaction reporting and pharmacovigilance: A nationwide survey
Source: Explor Res Clin Soc Pharm. 2025 Feb 18;18:100578. doi: 10.1016/j.rcsop.2025.100578 (PMC11919300; doi:10.1016/j.rcsop.2025.100578)
Supplement: Supplementary file 1 — Supplementary material 1 [file mmc1.docx]

**Supplementary material S1: Data collection tool measures and validity**

**Methods**

Item selection A total of 39 items were extracted from the literature after reviewing other studies related to the research subject [1-11]. The 39 items were subjected to internal review by the research team (three community pharmacists, and two clinical pharmacists) and external review by three experts in the field of community pharmacy to assess the appropriateness of the content and wording. This process reduced the number of items to 29 by removing items that were considered not relevant to community pharmacy practice (10 items). Using a consensus approach, the items were assessed in terms of relevance and representation of the construct being measured (i.e., content validity of the data collection tool). A Likert scale was chosen for scoring all items. Priori themes for the 29 items were set – Knowledge of community pharmacists about the drug-induced diseases (iatrogenic disease) (19 items), Attitude of community pharmacists toward pharmacovigilance and drug-induced disease (iatrogenic disease) reporting (6 items), and Practice of community pharmacists toward pharmacovigilance and ADR reporting (4 items).

Pretesting The data collection tool was piloted with five community pharmacists one by one with changes after each participant until no further changes were required. A cognitive interview and think-aloud interviews [12, 13] were used. Participants were asked for their feedback on the questions including the wording, content, necessity for addition or deletion, and format. This procedure of pretesting subjectively assessed face validity, i.e., whether the item appeared to measure what it was intended to measure.

Participant eligibility Registered pharmacists were eligible if they worked in a community pharmacy setting.

Participant recruitment and sample size Participants were recruited from community pharmacies through students who registered within the applied research in community pharmacy course at the Hashemite University in Jordan. The targeted sample size was estimated to be 123 based on the number of registered students.

Data management Online and paper-based questionnaires were distributed to eligible participants. Online questionnaires were created and distributed via a weblink using online survey software, Google Forms® from April to June 2023 for data collection. Google Forms^®^ is not GPPD compliant; however, the research team added a paragraph in the form which obviously explains what they will do with the collected data. Survey responses were collected anonymously to prevent IP address and location tracking. The analysis was performed using IBM-SPSS version 28.0 for Windows (IBM Corporation, Armonk, NY, USA).

Data analysis Validation and item reduction of the 29 items followed the procedure used by previous studies [14-17] and the statistical approach suggested by Field, 2013 [18] as follows:

Construct validity: A principal component analysis (PCA) was performed in an explorative manner using several statistics for item reduction and best fit the construct. The sampling adequacy for the PCA was checked using Kaiser–Meyer–Olkin (KMO) measure > 0.5. Bartlett’s test of sphericity was used to confirm the factorability of data (P < 0.05). The number of factors (PCA components) from the data was determined utilizing an extraction based on eigenvalue *>* 1. Varimax rotation was used to remove items with low loading (*<* 0.50) and cross-loading. Items with communalities extraction value *<* 0.50 were removed. Less than 50% non-redundant residuals with absolute values *>* 0.05 was considered goodness of fit. The multicollinearity was detected by the determinant of the R-matrix *<*10^-5^. Next, content validity was ensured through a discussion between a community pharmacist and the research team.

Reliability test: The final questionnaire was subjected to Cronbach’s alpha and composite reliability tests. In this study, the value of ≥ 0.70 in both tests was considered for inclusion of the factor and its item in the final questionnaire.

**RESULTS**

A total of 123 participants completed the survey. The vast majority of participants (91.9%) were aged 45 years or less and 57.7% were female. Of the 123 participants, most participants were from urban areas (92.7%). Just over half of participants (55.3%) had five years of experience or less (Table 1).

**Table 1.** Participants characteristics (n = 123).

| **Variable** | **Frequency** | **Percent (%)** |
| --- | --- | --- |
| **Age** |  |  |
| 21-25 years | 36 | 29.3 |
| 26-30 years | 42 | 34.1 |
| 31-35 years | 21 | 17.1 |
| 36-40 years | 8 | 6.5 |
| 41-45 years | 6 | 4.9 |
| 46-50 years | 6 | 4.9 |
| More than 50 years | 4 | 3.3 |
| **Gender** |  |  |
| Female | 71 | 57.7 |
| Male | 52 | 42.3 |
| **Place of pharmacy** |  |  |
| Urban areas | 114 | 92.7 |
| Rural areas | 9 | 7.3 |
| **Participant year of experience** |  |  |
| One year or less | 20 | 16.3 |
| 2-5 years | 48 | 39.0 |
| 6-9 years | 27 | 22.0 |
| 10 years or more | 28 | 22.8 |
|  | | |

There were 29 items included for analysis. No missing data were identified within participants’ responses. Items were removed due to reduced factor loading (n = 4), cross loading (n = 1), low communalities extraction (n = 1). The construct was best fitted with a total of 23 items structured into three themes, and these became the study questionnaire (Table 2).

Data presented in Table 2 indicate that the questionnaire has good construct validity. The three themes of the questionnaire in combination explained 66.64% of the variance. The determinant of the R- matrix of the questionnaire (5.2 × 10^-5^) suggests that the items do not have extreme multicollinearity and singularity. The nonredundant residuals with absolute values > 0.05 was 39%, which is low, indicating that the questionnaire had appropriate global goodness of fit. Moreover, the high KMO statistics (0.738) of the questionnaire suggested that the sample size was adequate, and significant, Bartlett’s test of sphericity (p < 0.001) indicated that the items were sufficiently correlated [15, 18].

The psychometric properties of the questionnaire indicate the questionnaire had good reliability based on the results from both the Cronbach’s alpha (0.772) and composite reliability (0.956) tests, demonstrating a high level of internal consistency for the questionnaire (Table 2). Cronbach’s alpha was > 0.8 for the knowledge theme. Whereas Cronbach’s alpha was ≥ 0.7 for the attitude and practice themes. The composite reliability was > 0.7 for all themes.

**Table 2.** Validation and reliability of study questionnaire (n = 123).

| **Item** | **MSA** | **Factor loading** | **Communalities** | **Cronbach’s Alpha** | **Composite reliability** |
| --- | --- | --- | --- | --- | --- |
| **Theme one:** **Knowledge of community pharmacists about the drug-induced diseases (iatrogenic disease)** | | | | | |
| 1-Gastritis can be induced by taking nonsteroidal anti-inflammatory drugs. | 0.637 | 0.678 | 0.637 | 0.814 | 0.928 |
| 2- Paralytic ileus can be induced by taking loperamide. | 0.629 | 0.721 | 0.629 |  |  |
| 3- Hypotension can be induced by taking ceftriaxone injection. | 0.654 | 0.640 | 0.654 |  |  |
| 4- Hyponatremia leading to ischemic heart disease can be induced by taking carbamazepine. | 0.648 | 0.733 | 0.648 |  |  |
| 5- Psychosis can be induced by taking methylprednisolone. | 0.578 | 0.680 | 0.578 |  |  |
| 6- Cognitive dysfunction can be induced by taking prednisolone. | 0.744 | 0.587 | 0.744 |  |  |
| 7- Parkinsonism can be induced by taking cinnarizine. | 0.620 | 0.621 | 0.620 |  |  |
| 8- Obesity can be induced by taking risperidone. | 0.724 | 0.709 | 0.724 |  |  |
| 9- Dyslipidemia can be induced by taking steroids (like estrogens and androgens). | 0.733 | 0.691 | 0.733 |  |  |
| 10- Menstrual dysfunction can be induced by taking valproic acid. | 0.726 | 0.739 | 0.726 |  |  |
| 11- Rhinitis can be induced by taking beta-blockers. | 0.581 | 0.607 | 0.581 |  |  |
| 12- Pruritis can be induced by taking angiotensin-converting enzyme inhibitors. | 0.712 | 0.767 | 0.712 |  |  |
| 13- Pruritis can be induced by taking statins. | 0.674 | 0.764 | 0.674 |  |  |
| 14- Osteoporosis can be induced by taking methotrexate. | 0.761 | 0.754 | 0.761 |  |  |
| **Theme two: Attitude of community pharmacists toward pharmacovigilance and drug-induced disease (iatrogenic disease) reporting** | | | | | |
| 1. I am satisfied that I have enough knowledge about drug-induced diseases. | 0.792 | 0.683 | 0.792 | 0.732 | 0.845 |
| 2. I am satisfied that I received sufficient education and training about drug-induced diseases. | 0.714 | 0.635 | 0.714 |  |  |
| 3. I am uncertain about recommending stopping the drug that I absolutely know its association with the reported problem by patient. | 0.620 | 0.710 | 0.620 |  |  |
| 4. As a community pharmacist, I have a responsibility to report ADR to JFDA. | 0.749 | 0.786 | 0.749 |  |  |
| 5. As a community pharmacist, I should only be required to consult the prescribing physician when a patient reports any problem associated to certain drug. | 0.620 | 0.788 | 0.620 |  |  |
| **Theme three: Practice of community pharmacists toward pharmacovigilance and ADR reporting** | | | | | |
| 1. Do you review patient’s medication list? | 0.736 | 0.722 | 0.736 | 0.700 | 0.778 |
| 2. If patients tell you about symptoms occur with them, do you ask them about their medication list? | 0.652 | 0.691 | 0.652 |  |  |
| 3. When a new medication is introduced to the market, do you ask patients who are taking this medication if they experienced any side effect from it? | 0.747 | 0.782 | 0.747 |  |  |
| 4. During your professional career, do you record the reported ADR from patients on patients’ medical records? | 0.792 | 0.526 | 0.792 |  |  |
| ADR, adverse drug reaction |  |  |  |  |  |

**References**

1. Aggarwal A, Sharma M, Maisnam I, Ghosh S, Aggarwal S, Bhattacharya S, et al. Drug-induced bone disorders: a systematic review. Indian Journal of Rheumatology. 2019;14(Suppl 1):S44-S51.

2. Tandon VR, Khajuria V, Mahajan V, Sharma A, Gillani Z, Mahajan A. Drug-induced diseases (DIDs): An experience of a tertiary care teaching hospital from India. The Indian Journal of Medical Research. 2015;142(1):33.

3. Siraj J, Shafi M, Ejeta F, Feyisa D, Kebede O, Hassen S. Willingness, Attitude, and Associated Factors towards Adverse Drug Reaction Reporting among Healthcare Providers in Mizan Tepi University Teaching Hospital, Southwest Ethiopia. Advances in Pharmacological and Pharmaceutical Sciences. 2022;2022.

4. Albayrak A, Karahalil B. Pharmacist’s Knowledge and Behaviors Toward Pharmacovigilance and Adverse Drug Reactions Reporting Process in Türkiye. Turkish Journal of Pharmaceutical Sciences. 2022;19(6):694.

5. Reich A, Ständer S, Szepietowski JC. Drug-induced pruritus: a review. Acta dermato-venereologica. 2009;89(3):236-44.

6. López-Pintor E, Lumbreras B. Use of gastrointestinal prophylaxis in NSAID patients: a cross sectional study in community pharmacies. International Journal of Clinical Pharmacy. 2011;33:155-64.

7. Shraim NY, Al Taha TA, Qawasmeh RF, Jarrar HN, Shtaya MA, Shayeb LA, et al. Knowledge, attitudes and practices of community pharmacists on generic medicines in Palestine: a cross-sectional study. BMC health services research. 2017;17:1-9.

8. Rizvi SF, Nawaz H, Fatima A. The Prevalence of Drug Induced Gastritis among Patients with ADR. PAKISTAN JOURNAL OF MEDICAL & HEALTH SCIENCES. 2018;12(2):967-9.

9. Intravooth T, Staack AM, Juerges K, Stockinger J, Steinhoff BJ. Antiepileptic drugs-induced hyponatremia: Review and analysis of 560 hospitalized patients. Epilepsy research. 2018;143:7-10.

10. Lovell AR, Ernst ME. Drug-induced hypertension: focus on mechanisms and management. Current hypertension reports. 2017;19:1-12.

11. Krishnan N, Kasthuri A. Iatrogenic disorders. Medical journal, Armed Forces India. 2005;61(1):2.

12. DeMuro CJ, Lewis SA, DiBenedetti DB, Price MA, Fehnel SE. Successful implementation of cognitive interviews in special populations. Expert review of pharmacoeconomics & outcomes research. 2012;12(2):181-7.

13. Wolcott MD, Lobczowski NG. Using cognitive interviews and think-aloud protocols to understand thought processes. Currents in Pharmacy Teaching and Learning. 2021;13(2):181-8.

14. El-Den S, Schneider C, Mirzaei A, Carter S. How to measure a latent construct: Psychometric principles for the development and validation of measurement instruments. International Journal of Pharmacy Practice. 2020;28(4):326-36.

15. Mirzaei A, Carter SR, Chen JY, Rittsteuer C, Schneider CR. Development of a questionnaire to measure consumers’ perceptions of service quality in community pharmacies. Research in Social and Administrative Pharmacy. 2019;15(4):346-57.

16. Shrestha S, Poudel A, Reeve E, Linsky AM, Steadman KJ, Nissen LM. Development and validation of a tool to understand health care professionals' attitudes towards deprescribing (HATD) in older adults with limited life expectancy. Research in Social and Administrative Pharmacy. 2022;18(9):3596-601.

17. GÜVENDİR MA, ÖZKAN YÖ. Item removal strategies conducted in exploratory factor analysis: A comparative study. International Journal of Assessment Tools in Education. 2022;9(1):165-80.

18. Field A. Discovering statistics using IBM SPSS statistics: sage; 2013.
