# Supplementary material for: Knowledge, attitude, and practice among community pharmacists toward adverse drug reaction reporting and pharmacovigilance: A nationwide survey
Source: Explor Res Clin Soc Pharm. 2025 Feb 18;18:100578. doi: 10.1016/j.rcsop.2025.100578 (PMC11919300; doi:10.1016/j.rcsop.2025.100578)
Supplement: Supplementary file 2 — Supplementary material 2 [file mmc2.docx]

SUPPLEMENTARY MATERIAL S2. STUDY QUESTIONNAIRE.

**Participant information about the study**

**Study title:** Knowledge, attitude, and practice among community pharmacists toward pharmacovigilance and adverse drug reaction reporting: A cross‑sectional survey

You are invited to participate in a research study. Before deciding whether you would like to participate, please take the time to read this information. It is important that you understand why this study is being conducted and what you will be asked to do if you agree to participate. If anything is unclear, or if you would like further information, please contact the principal investigator (see below). All your information will be treated confidentially.

**What is the purpose of this study?**

Adverse drug reaction (ADR) has been defined as “*an appreciably harmful or unpleasant reaction, resulting from an intervention related to the use of a medicinal product, which predicts hazard from future administration and warrants prevention or specific treatment, or alteration of the dosage regimen, or withdrawal of the product*”. ADRs is considered an important health problem which burdens people since it increases the risk of hospitalization, mortality and morbidity and increases health-related cost. Pharmacovigilance is detection, assessment, understanding and prevention of ADR. Awarenesses about pharmacovigilance will enhance medications safety profiles and ensure better efficacy of them. Thus, we need research to determine the pattern of reporting of adverse drug event in routine practice, as a first step to develop Good Pharmacovigilance Practice programs. Therefore, the current study aims understand the attitudes and behaviors of community pharmacists towards adverse drug reactions in community pharmacy sector.

**Why have I been chosen to take part?**

You have been approached to take part in this study because you are a pharmacist who holds at least a bachelor’s degree in pharmacy or Doctor of Pharmacy, currently practicing the profession of community pharmacist, registered in the Jordan Pharmacists Syndicate, and able to sign the consent form.

**Is my participation voluntary?**

Yes, your participation in the questionnaire survey is entirely voluntary. You can withdraw anytime you wish during the survey, until the point you return the survey to us.

**What will happen to the data?**

All your information will remain confidential, and you will not be asked for any personal information, such as your name or date of birth. If you decide to participate in the study, you will receive its own questionnaire to fill out, either in paper or electronic form, as you wish.

**You are free to discuss your participation in this study with the principal investigator:**

Assistant Professor at Faculty of Pharmaceutical Sciences, The Hashemite University.

Mobile: 00962775919109, Email: [alwidyantahani@hu.edu.jo](mailto:alwidyantahani@hu.edu.jo).

**Participant study ID:** ………………………….

**Participant Consent Form**

**Study title:** Knowledge, attitude, and practice among community pharmacists toward pharmacovigilance and adverse drug reaction reporting: A cross‑sectional survey

|  | Please initials  box |
| --- | --- |
| 1. I have read and understood the nature of the study, its objectives, how it will be conducted, its benefits, and the potential risks of participating in it. |  |
| 2. I understand that my participation is voluntary and that I am free to withdraw at any time, without giving any reason. |  |
| 3. I have had the opportunity to consider the information, ask questions and have had these answered satisfactorily. |  |
| 4. I understand that what is discussed during the completion of questionnaire is confidential. |  |
| 5. I understand that direct quotations from my answers may be reproduced in reports and papers, but that confidentiality and anonymity will be maintained, and it will not be possible to identify me in any publications or presentations |  |
| 6. I understand that my personal information (including consent forms) will be held securely in the School of Pharmacy, The Hashemite University. |  |
| 7. I agree to take part in the above study. |  |

| ____________________  Name of Participant  (Please print) | _______________  Date | Signature |
| --- | --- | --- |
| _____________________  Name of Participant  (Please print) | _______________  Date | Signature |

**Knowledge, attitude, and practice among community pharmacists toward pharmacovigilance and adverse drug reaction reporting: A cross‑sectional survey**

Please Answer all the following questions in this questionnaire which consists of four parts.

**Section 1: Demographic and pharmacy related information.**

**In answering the questions (1-17) below, please follow the following directions:**

1. Answer all questions.
2. Choose one answer for each question.
3. Where you choose other as your answer, please specify.

| **1- Age** |  |
| --- | --- |
| a. less than 21 years. | b. 21-25 years. |
| c. 26-30 years. | d. 31-35 years. |
| e. 36-40 years. | f. 41-45 years. |
| g. 46-50 years. | h. 51 years or more. |
|  |  |
| **2- Gender** |  |
| a. Male. | b. Female. |
|  |  |
| **3- Year(s) of experience** |  |
| a. One year or less. | b. 2-5 years. |
| c. 6-9 years. | d. 10 years or more. |
|  |  |
| **4- Degree** |  |
| 1- Pharmacy. | 2- Doctor of pharmacy. |
|  |  |
| **5- Educational level** |  |
| a. Bachelor’s degree. | b. Master’s degree. |
| c. PhD degree. |  |
|  |  |
| **6- Place of pharmacy** |  |
| a. Urban area (i.e., city, town). | b. Rural area (i.e., village). |
|  |  |
| **7- Area around the pharmacy** |  |
| a. Near a hospital. | b. Near medical center. |
| c. Near medical clinic(s). | d. No medical facilities around it. |
| **Section 2: Awareness about pharmacovigilance and ADR reporting** | |
| **1- Do you think that adverse drug reactions could be monitored by the community pharmacist?** | |
| a. Yes. | b. No. |
|  |  |
| **2- Do you think that adverse drug reactions should be reported by the community pharmacist?** | |
| a. Yes. | b. No. |
|  | |
| **3- Do you report suspected adverse drug reactions in your community setting?** | |
| a. Yes. | b. No. |
|  |  |
| **4- If your answer to question 10 above is Yes, I report adverse drug reactions because:** | |
|  |  |
|  |  |
|  |  |
|  | |
|  |  |
| **5- During your professional career, how many times have you reported adverse drug reactions?** | |
| a. Not applicable, I do not report adverse drug reactions. | b. 5 times and less. |
| c. 6-10 times. | d. 11-15 times. |
| e. 16-20 times. | f. 21 times and more. |
|  |  |
| **6- How do you report the adverse drug reactions?** | |
| a. Not applicable, I do not report adverse drug reactions. | b. I phone the drug company. |
| c. I tell the representative of the drug company. | d. I fill the adverse drug reaction reporting form. |
| e. Others, please specify …………………. | |
|  |  |
| **7- If your answer to question 10 above is No, I do not report adverse drug reactions because:** | |
|  |  |
|  |  |
|  |  |
|  |  |
|  |  |
|  | |
|  |  |
| **8- What is pharmacovigilance?** | |
| a. The reporting of adverse drug reactions. | |
| b. Detection, assessment, understanding, and prevention of adverse drug reactions. | |
| c. I’m not sure but I could choose option a. | d. I’m not sure but I could choose option b. |
| e. I don’t know. |  |
|  |  |
| **9- Are you aware of the pharmacovigilance center in Jordan?** | |
| a. Yes. | b. No. |
|  |  |
| **10- The healthcare professional responsible for reporting adverse drug reactions is:** | |
| a. Physician. | b. Hospital pharmacist |
| c. Community pharmacist. | d. Clinical pharmacist. |
| e. Nurse. | f. Any health care professionals. |
| g. Patients. | h. I do not know. |

**Section 3: Community pharmacist’s knowledge about** **drug-induced diseases (iatrogenic disease).**

**In answering the questions (1-14) below, please follow the following directions:**

1. Answer all questions.
2. For each statement in the table below, select one option from the three Likert scale:
3. Yes. b. No. c. I do not know.

| 1. Gastritis can be induced by taking nonsteroidal anti-inflammatory drugs. | Yes   | No   | I do not know.  |
| --- | --- | --- | --- |
| 2. Paralytic ileus can be induced by taking loperamide. | Yes   | No   | I do not know.  |
| 3. Hypotension can be induced by taking ceftriaxone injection. | Yes   | No   | I do not know.  |
| 4. Hyponatremia leading to ischemic heart disease can be induced by taking carbamazepine. | Yes   | No   | I do not know.  |
| 5. Psychosis can be induced by taking methylprednisolone. | Yes   | No   | I do not know.  |
| 6. Cognitive dysfunction can be induced by taking prednisolone. | Yes   | No   | I do not know.  |
| 7. Parkinsonism can be induced by taking cinnarizine. | Yes   | No   | I do not know.  |
| 8. Obesity can be induced by taking risperidone. | Yes   | No   | I do not know.  |
| 9. Dyslipidaemia can be induced by taking steroids (like estrogens and androgens. | Yes   | No   | I do not know.  |
| 10. Menstrual dysfunction can be induced by taking valproic acid. | Yes   | No   | I do not know.  |
| 11. Rhinitis can be induced by taking beta-blockers. | Yes   | No   | I do not know.  |
| 12. Pruritis can be induced by taking angiotensin-converting enzyme inhibitors. | Yes   | No   | I do not know.  |
| 13. Pruritis can be induced by taking statins. | Yes   | No   | I do not know.  |
| 14. Osteoporosis can be induced by taking methotrexate. | Yes   | No   | I do not know.  |

**Section 4: Attitude-related questions.**

**In answering the questions (1-5) below, please follow the following directions:**

1. Answer all questions.
2. For each statement in the table below, select one option from the five Likert scale:
3. Strongly agree. b. Agree. c. Neutral. d. Disagree. e. Strongly disagrees.

| 1. I’m satisfied that I have enough knowledge about drug-induced diseases. | Strongly agree   | Agree   | Neutral   | Disagree   | Strongly disagree   |
| --- | --- | --- | --- | --- | --- |
| 2. I’m satisfied that I received sufficient education and training about drug-induced diseases. | Strongly agree   | Agree   | Neutral   | Disagree   | Strongly disagree   |
| 3. I am uncertain about recommending stopping the drug that I absolutely know its association with the reported problem by the patient. | Strongly agree   | Agree   | Neutral   | Disagree   | Strongly disagree   |
| 4. As a community pharmacist, I have a responsibility to report adverse drug reactions to the Jordanian Food and Drug Administration. | Strongly agree   | Agree   | Neutral   | Disagree   | Strongly disagree   |
| 5. As a community pharmacist, I should only be required to consult the prescribing physician when a patient reports any problem associated to certain drug. | Strongly agree   | Agree   | Neutral   | Disagree   | Strongly disagree   |

**Section 5: Practice-related questions.**

**In answering the questions (1-4) below, please follow the following directions:**

1. Answer all questions.
2. For each statement in the table below, select one option from the three Likert scale:
3. Always. b. Sometimes. c. Never.

| 1. Do you review patient’s medication list? | Always   | Sometimes   | Never   |
| --- | --- | --- | --- |
| 2. If patients tell you about symptoms occur with them, do you ask them about their medication list? | Always   | Sometimes   | Never   |
| 3. When a new medication introduced to the Jordanian market, do you ask patients who are taking this medication if they experienced any side effect from it? | Always   | Sometimes   | Never   |
| 4. During your professional career, do you record the reported adverse drug reactions from patients on patients’ medical records? | Always   | Sometimes   | Never   |

**Thank you for considering participation in this study.**
